# Therapies of varicose veins: Protocol for the reporting and methodological quality of pairwise meta-analyses

**DOI:** 10.1097/MD.0000000000016042

**Published:** 2019-06-21

**Authors:** Jie Ding, XiaoFei Mu, Yuan Yuan, LiYao Tang, KongXi Wei, XiYun Zhao, LiNa Qing, Cai Liu

**Affiliations:** a Chinese Medicine Hospital, Occupation University of Wuwei; b Wuwei People's Hospital, Wuwei; c Gansu University of Traditional Chinese Medicine; d The 94th Hospital of the Joint Logistic Support Force of the Chinese People's Liberation Army; e Affiliated Hospital of Gansu University of Traditional Chinese Medicine; f The First Hospital of Lanzhou University; g The Third People's Hospital of Gansu Province, Lanzhou, China.

**Keywords:** AMSTAR-2, evidence mapping, meta-analyses, overview, PRISMA, varicose veins

## Abstract

**Background:**

Many pairwise meta-analyses (MAs) related to therapies of varicose veins have been published, but their reporting and methodological quality remain unclear. The present study was designed to assess the overall quality of pairwise MAs related to therapies of varicose veins.

**Methods:**

We will systematically search 4 electronic databases, including PubMed, EMBASE, Cochrane Library, Chinese Biomedical Database, to identify pairwise MAs related to therapies of varicose veins. The search time-span was set from inception to March 2019. The pairwise MAs related to therapies of varicose veins will be included in our overview. The reporting and methodological quality of included MAs will be assessed using preferred reporting items for systematic review and meta-analysis and a measurement tool to assess systematic reviews 2, respectively. Meanwhile, we will extract some general characteristics of included MAs, including first author; published year, journal, sample size, number of studies, number of randomized controlled trials and intervention details, and so on. All literatures screening, quality assessment, and data extraction will be independently completed by 2 of all reviewers, and any disagreement will be resolved by discussion. Besides, an increasingly popular method – evidence mapping, will be used to present the whole evidence landscape related to therapies of varicose veins. The assessment results will be presented as percentage and event/total. The Excel 2016 will be used to manage and analyze data.

**Results:**

The results of the overview will be submitted to a peer-reviewed journal for publication.

**Conclusion:**

This overview will summarize the overall reporting and methodological quality related to therapies of varicose veins.

**PROSPERO registration number:**

CRD42019126722.

## 1 Introduction

Varicose vein is one of the most commonly chronic venous diseases, and prevalence for varicose veins is higher, 1% to 73% in females and 2% to 56% in males in Western countries.^[1]^ A commonly used diagnose criteria for varicose veins: dilated, palpable subcutaneous veins generally larger than 4 mm.^[2]^ Although some patients with varicose veins remain asymptomatic, others may experience pain, aching, heaviness, and itching, that can seriously affect quality of life.^[3]^ It also poses big financial burden; a recent report shows that approximately 2% of national healthcare resources was spent on treatment of varicose veins in London.^[4]^ In practice, varicose vein treatments including conservative therapy, surgery involving saphenous ligation and stripping, and others new treatments.^[5,6,7,8]^ Until recently, surgery was considered as standard treatment, it also with high ligation and stripping to knee level, and combing with phlebectomies.^[9,10,11]^ However, operation may occasionally be associated with significant postoperative morbidity, including bleeding, thrombophlebitis and groin infection, and so on.^[8]^

The meta-analyses (MAs) is a review that always using systematic approaches to identify, select and critically appraise primary studies. And many pairwise MAs^[12,13,14]^ related to therapies of varicose veins have been published, but their reporting and methodological quality remain unclear. However, the reporting and methodological quality of MAs very important for clinicians and clinical practice guideline developers to judge their reliability. Therefore, the guideline – preferred reporting items for systematic reviews and meta-analyses (PRISMA), which was developed and as a checklist was recommended to report MAs.^[15]^ And a well-known tool – a measurement tool to assess systematic reviews (AMSTAR),^[16]^ which was developed and recommended to assess the methodological quality of MAs or as a methodology guideline for conducting of MAs, and it was upgraded to AMSTAR-2 in 2017.^[17]^

To the best of our knowledge, pairwise MAs related to therapies of varicose veins has not been apprised using PRISMA and AMSTAR-2. Therefore, the present study was designed to assess the overall quality of pairwise MAs related to therapies of varicose veins, and a recent popular method – evidence mapping (EM),^[18,19]^ will be used to present the whole evidence landscape.

## 2 Methods

This protocol follows PRISMA-P,^[20]^ and the reporting of this study results will follow PRISMA guideline.^[15]^ This overview has been registered on the international prospective register of systematic reviews.

### 2.1 Eligibility criteria

We will include pairwise MAs related to therapies of varicose veins, and they should be published in Chinese or English. Some publication document types will not be considered, such as letter, comment, and conference abstract, and so on. If there are duplicated papers for 1 special research, we will include the latest one and could provide the most information on quality of the research.

### 2.2 Search strategy

We plan to search the following databases to identify pairwise MAs related to therapies of varicose veins from inception to March 2019: PubMed, EMBASE, The Cochrane Library, Chinese Biomedical Database. Language will be restricted as English or Chinese. The search strategy of PubMed was as:

#1 “Varicose Veins” [Mesh]
#2 “chronic lower extremity venous insufficiency” [Title/Abstract]) OR “varicose veins” [Title/Abstract]) OR “varicose vein” [Title/Abstract]) OR “veins varicose” [Title/Abstract]) OR “vein varicose” [Title/Abstract]) OR Varix∗ [Title/Abstract]) OR Varice∗ [Title/Abstract]) OR varicosi∗ [Title/Abstract]) OR “enlarged twisted veins” [Title/Abstract]) OR “tortuous veins” [Title/Abstract]) OR “saphenous vein” [Title/Abstract]) OR “saphenous veins” [Title/Abstract]) OR “varicose venous” [Title/Abstract]
#3 #1 OR #2
#4 “Systematic Reviews as Topic” [Mesh] OR “Systematic Review” [Publication Type] OR “Meta-Analysis as Topic” [Mesh] OR “Meta-Analysis” [Publication Type]
#5“systematic review” [Title/Abstract] OR “systematic reviews” [Title/Abstract] OR “meta analysis” [Title/Abstract] OR “meta analyses” [Title/Abstract] OR metanalysis [Title/Abstract] OR metanalyses [Title/Abstract] OR meta-analysis [Title/Abstract] OR meta-analyses [Title/Abstract]
#6 #4 OR #5
#7 #3 AND #6

### 2.3 Study selection

**Figure 1:**
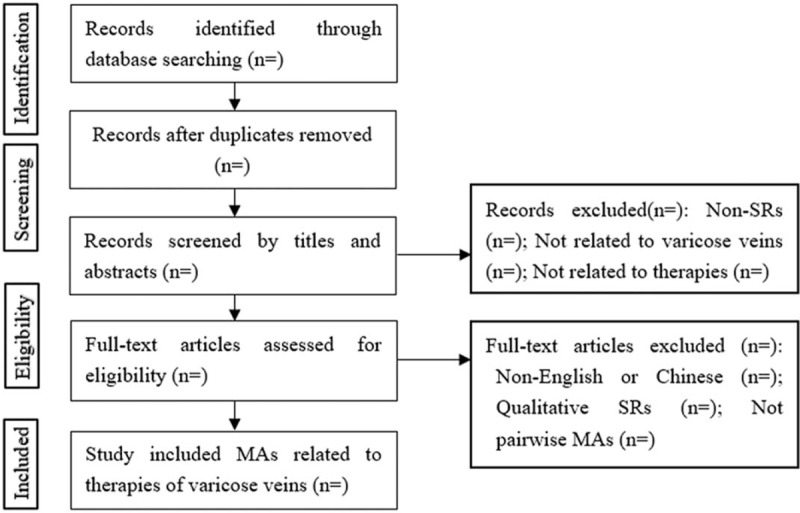
Selecting flowchart of meta-analyses related to therapies of varicose veins.

Initial records from electronic databases will be imported into the EndNote X8 software. First, the titles and abstracts of all records will be reviewed independently by 2 reviewers. Then, full text of all potentially relevant MAs will be retrieved to make the final decision. Any conflict will be resolved by discussion. A flow diagram will be used to describe the process of MAs selection (Fig. 1).

### 2.4 Quality assessment

#### 2.4.1 Reporting quality assessment

The PRISMA checklist including 7 parts with 27 items.^[15]^ The developers of the PRISMA proposed 3 answer options for each item: yes, no and partial. We will use PRISMA checklist to assess each item of the included MAs. Finally, we will count the percentage and event of MAs who answer “yes,” who answer “partial” and who answer “no” for each item. The assessed result will be presented according to the form of Table 1.

**Table 1:**
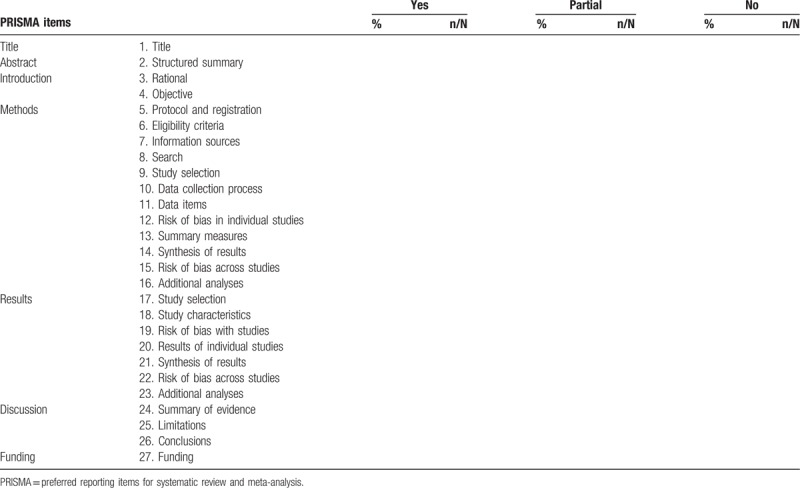
PRISMA assessed results of meta-analyses related to therapies of varicose veins.

#### 2.4.2 Methodological quality assessment

Two independent reviewers will assess the methodological quality of included MAs using the AMSTAR-2 tool,^[17]^ which consists of 16 items. In our study, we will take AMSTAR-2 to assess each included MA and count the percentage and event who answer “yes,” “partial yes” and “no” for each item. The overall confidence on the results of the MAs will be rated according to the following 4 categories: High (no or 1 noncritical weakness: the MA provides an accurate and comprehensive summary of the results of the available studies that address the question of interest), Moderate (more than 1 noncritical weakness: the MA has more than 1 weakness but no critical flaws), Low (1 critical flaw with or without noncritical weaknesses: the MA has a critical flaw and may not provide an accurate and comprehensive summary of available studies that address the question of interest), Critically low (more than 1 critical flaw with or without noncritical weakness: the MAs has more than 1 critical flaw and should not be relied on to provide an accurate and comprehensive summary of the available studied). Finally, we will count the percentage and event of each items, and categories of the overall confidence on the included MAs (details will be shown a table according to the form of Table 2).

**Table 2:**
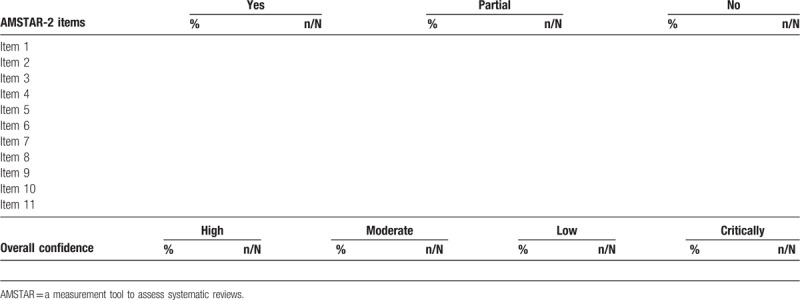
AMSTAR-2 assessed results of meta-analyses related to therapies of varicose veins.

### 2.5 Evidence mapping

We will use EM method to present the evidence landscape related to therapies of varicose veins. The presentation form of EM will be using the bubble plot and, which will display information on 4 dimensions: AMSTAR-2 assessment (*y*-axis); the rating of authors’ conclusions (including beneficial, probably beneficial, harmful, no differential effect, and inconclusive) of the MAs (*x*-axis), the number of primary studies included in each MA (bubble size), and a pie will be used to show the proportion of randomized controlled trials.

### 2.6 Data extraction and synthesis

The Excel 2016 (Microsoft Corp, WA) form will be used to extract and analyze data from the included MAs. Extracted information will include: first author; published year; journal; funding; sample size; number of studies; number of studies; details of interventions and control conditions; effect size and confidence interval; outcomes; conclusions and contents related to PRISMA and AMSATR-2. And the assessment results and other data will be presented as percentage or (and) event/total.

### 2.7 Ethics and dissemination

Because this study is not a clinical study, and we will search and evaluate only existing sources of literature. So ethical approval is not required.

## 3 Discussion

To the best of our knowledge, this is the first overview to assess overall quality of pairwise MAs related to therapies of varicose veins by using PRISMA and AMSATR-2. Meanwhile, a popular method-EM, will be used to present evidence. And we believe the results of this overview will provide some references for clinicians and clinical practice guideline developers.

## Author contributions

Jie Ding, XiaoFei Mu, and Cai Liu conceived the study. XiYun Zhao and LiNa Qing designed the flowchart of literature selection and 2 tables. Yuan Yuan and KongXi Wei designed the search strategy. Yuan Yuan and LiYao Tang participated critical revision of the manuscript. All the authors contributed to draft and approve the study protocol. Jie Ding will supervise the overall conduct of the study.

**Conceptualization:** Jie Ding, XiaoFei Mu, Cai Liu.

**Investigation:** Yuan Yuan, LiYao Tang, KongXi Wei.

**Methodology:** XiYun Zhao, LiNa Qing.

**Resources:** Yuan Yuan.

**Writing – original draft:** Jie Ding.

**Writing – review and editing:** Jie Ding, Yuan Yuan, Cai Liu.
